# Supplementary material for: Factors affecting job performance of Sri Lankan IT professionals working from home
Source: PLoS One. 2023 Dec 20;18(12):e0295305. doi: 10.1371/journal.pone.0295305 (PMC10732380; doi:10.1371/journal.pone.0295305)
Supplement: S1 Appendix — (DOCX) [file pone.0295305.s001.docx]

**S1 Appendix. Questionnaire.**

**1^st^ of June 2022**

**A RESEARCH ON THE EFFECTS OF WORKING FROM HOME POLICIES ON THE PERFORMANCE OF IT PROFESSIONALS IN SRI LANKA**

A RESEARCH ON THE EFFECTS OF WORKING FROM HOME POLICIES ON THE PERFORMANCE OF IT PROFESSIONALS IN SRI LANKA

Dear Respondents to the Survey,

At SLIIT School of Business, I'm a postgraduate student studying business administration. The purpose of this survey is to gather information that will be used to study the numerous elements that influence employee performance in the work-from-home environment.

Your participation in this survey would be very appreciated by me as a postgraduate student at the SLIIT School of Business, as it will assist me in completing my Research Report by providing your honest opinion and experience. All information submitted will be 'Anonymous,' will be kept with the highest confidentiality, and will be used only for the purpose for which it was provided.

I appreciate you taking the time to read this and thank you in advance for your important time.

**Section A – Demographic Factor**

Please select the most suitable answer for each statement.



1. Age 20 – 25 years 26 – 30 years 31 – 40 years



41 – 50 years > 51 years



1. Gender Male Female



1. Education GCE A/L Diploma Undergraduate



Graduate Postgraduate

1. Are you an IT Professional based in Sri Lanka?

    Yes No
2. What is your current Designation Intern Engineer

Senior Engineer Technical Lead Architect and above

1. How long have you been working in your **current** working place?



Less than 6 months 6-24 months



2-5 years more than 5 years

1. What is the name of your organization?


2. How large is your organization?

Less than 10 employees Less than 50 employees

50-200 employees More than 200 employees

1. Civil status Single Married



9.1 If married, does the spouse do a job?



Yes No



9.2 Do you have children?



Yes No



1. Do you use a virtual office platform (work from home) or a traditional office platform (work from office) to execute your work?

Virtual office platform Traditional office platform

Both platforms

1. Did you practice WFH after the onset of the COVID-19 pandemic and the stay-at-home request from the government?

Yes No



1. Suppose your productivity in the workplace is 100%, how do you evaluate your work productivity at home? Please answer this question considering all your tasks.

- 1 - Between 0%-49%
- 2 - Between 50%-99%
- 3 - 100%. No Change
- 4 - Between 101%-150%
- 5 -More than 150%

1. I have flexible working hours/flexible work schedules at my workplace

Yes No



1. On average, how much time daily did you spend in online meetings

- None
- 30 minutes or less per day
- More than 30 minutes but less than 2 hour per day
- More than 2 hours but less than 4 hours per day
- 4 or more hours per day

1. I connect with my team

- Every 2 hours
- Twice a day
- Once a day
- Once in 2 days
- Once in a week
- Other
- N/A

**Section B**

Please select (×) the most appropriate term (strongly Disagree to strongly agree) for each question below.

| Strongly Disagree | Agree | Neutral | Disagree | Strongly Agree |
| --- | --- | --- | --- | --- |
| 1 | 2 | 3 | 4 | 5 |

| **Q.No** | **Question** | **1** | **2** | **3** | **4** | **5** |
| --- | --- | --- | --- | --- | --- | --- |
|  | **Productivity in WFH** |  |  |  |  |  |
| ***16P | Poor telecommunication environment at home relative to the workplace affects negatively to my productivity | **1** | **2** | **3** | **4** | **5** |
| 17P | Rules and regulations that require some tasks to be conducted in the office affects negatively to my productivity | **1** | **2** | **3** | **4** | **5** |
| ***18P | I have some tasks cannot be conducted at home even though these are not required by the rules and regulations and must go to office. | **1** | **2** | **3** | **4** | **5** |
| ***19P | It is difficult to concentrate on the job because of the presence of family members | **1** | **2** | **3** | **4** | **5** |
| ***20P | Lack of a private room specifically designed for work workplace affects negatively to my productivity | **1** | **2** | **3** | **4** | **5** |
| ***21P | Loss of immediate communication that is only possible through face-to-face interactions with colleagues at the workplace affects negatively to my productivity. | **1** | **2** | **3** | **4** | **5** |
| 22P | Lack of pressure from boss, colleagues, and subordinate affects positively to my productivity | **~~1~~** | **~~2~~** | **~~3~~** | **~~4~~** | **~~5~~** |
| ***23P | Inadequate facilities like Internet, Electricity, A/C etc affects negatively to my productivity | **1** | **2** | **3** | **4** | **5** |
|  | **Communication** |  |  |  |  |  |
| 24C | I feel informed & supported by my organization | **1** | **2** | **3** | **4** | **5** |
| 25C | I feel included in our team decisions. | **1** | **2** | **3** | **4** | **5** |
| 26C | I feel connected enough to my teammates | **1** | **2** | **3** | **4** | **5** |
| 27C | I feel comfortable talking with my colleagues through the internet. | **1** | **2** | **3** | **4** | **5** |
| 28C | I don't feel alienated or abandoned when I work from home. | **1** | **2** | **3** | **4** | **5** |
| 29C*** | I feel unscheduled ad hoc meetings lead to disrupt the work I do and my productivity. | **1** | **2** | **3** | **4** | **5** |
| 30C | My supervisor gives me feedback on my performance that I find helpful. | **1** | **2** | **3** | **4** | **5** |
| 31C | I am very satisfied with respect to the length of the online meetings | **1** | **2** | **3** | **4** | **5** |
| 32C *** | I feel too many calls / meetings scheduled in a day gives me less time to focus on my work. | **1** | **2** | **3** | **4** | **5** |
|  | **Flexible work arrangement** |  |  |  |  |  |
| 33FW | I feel I am more productive and less stressed when my work arrangement is flexible. | **1** | **2** | **3** | **4** | **5** |
| 34FW | I have the flexibility I need to manage my work and non-work interests e.g., caring responsibilities, study, sporting interests etc. | **1** | **2** | **3** | **4** | **5** |
| 35FW*** | It is difficult for me to adopt a flexible working arrangement because of a lack of support from my supervisor | **1** | **2** | **3** | **4** | **5** |
| 36FW | I find it easier to manage my work when there are less ad-hoc meetings and more pre scheduled calls in my calendar. | **1** | **2** | **3** | **4** | **5** |
| 37FW*** | I feel there is an impact on depression & distress due to virtual office work | **1** | **2** | **3** | **4** | **5** |
| 38FW | Due to the virtual office platform, it saves a lot of time consumed for transportation which helps me utilize in other work | **1** | **2** | **3** | **4** | **5** |
| 39FW | Virtual work has a huge responsibility than regular work | **1** | **2** | **3** | **4** | **5** |
| 40FW*** | I feel virtual office hours last longer than your regular working hours |  |  |  |  |  |
| 41FW | It doesn’t affect me If flexible work arrangements lead me to work on weekends and other holidays. |  |  |  |  |  |
|  | **Work Life Balance** |  |  |  |  |  |
| 42WL | My work life balance has been changed for the better after working from home policies were enforced. | **1** | **2** | **3** | **4** | **5** |
| 43WL*** | I feel that my family life influences my ability to perform work responsibilities | **1** | **2** | **3** | **4** | **5** |
| 44WL*** | My workload has been increased in terms of the household responsibilities due to remote working | **1** | **2** | **3** | **4** | **5** |
| 45WL | I am able to disconnect from digital devices outside working hours/after completing the assigned work tasks | **1** | **2** | **3** | **4** | **5** |
| 46WL | There is a positive impact of virtual office on family satisfaction | **1** | **2** | **3** | **4** | **5** |
| 47WL | Normally I spend sufficient time enhancing my physical health due to virtual office | **1** | **2** | **3** | **4** | **5** |
| 48WL | I have social value and respect due to my job role and position | **1** | **2** | **3** | **4** | **5** |
| 49WL | I am satisfied with my job role and the duties I get to fulfill working from home. | **1** | **2** | **3** | **4** | **5** |
| 50WL*** | When virtual working days are increasing, it indirectly affects the free time that leaves to spend with the family members | **1** | **2** | **3** | **4** | **5** |
|  | **Working Environment** |  |  |  |  |  |
| 51WE | I have an adequate level of isolation and a space to work from home without any disturbances. | **1** | **2** | **3** | **4** | **5** |
| 52WE | I have adequate lighting in my home working space to fulfill my job responsibilities | **1** | **2** | **3** | **4** | **5** |
| 53WE | I have minimum noise levels around your home working space which doesn’t let me lose focus of my job responsibilities | **1** | **2** | **3** | **4** | **5** |
| 54WE | I have required resources to perform my daily tasks (The Internet service/Software (programs) etc.) | **1** | **2** | **3** | **4** | **5** |
| 55WE | Working alone and without supervision has had no detrimental influence on my performance. | **1** | **2** | **3** | **4** | **5** |
| 56WE | I never get stressed due to my work and the environment surrounding me. | **1** | **2** | **3** | **4** | **5** |
| 57WE*** | I feel virtual office platform may lead to psychological problems in the future | **1** | **2** | **3** | **4** | **5** |
| 58WE*** | Due to a lack of time outside of work, I am less involved in social gatherings/ friends | **1** | **2** | **3** | **4** | **5** |

**NOTE**

The answers for questions marked with ***, are reversed in descending order when analyzing.
