# Supplementary material for: Factors affecting job performance of Sri Lankan IT professionals working from home
Source: PLoS One. 2023 Dec 20;18(12):e0295305. doi: 10.1371/journal.pone.0295305 (PMC10732380; doi:10.1371/journal.pone.0295305)
Supplement: Attachment — Submitted filename: Response to Reviewers.docx [file pone.0295305.s003.docx]

**Point by point response to editor and reviewers**

Dear editor and the reviewers,

We would like to express our profound appreciation to the editor and the reviewers for the valuable comments and suggestions made on our manuscript which were very helpful in revising and improving it.

Please note that the line numbers referred in this document is aligned with the revised manuscript which has track changes.

| **Comments of the Reviewer** | **Comments of the Authors** |
| --- | --- |
| 1. Is the manuscript technically sound, and do the data support the conclusions?  The manuscript must describe a technically sound piece of scientific research with data that supports the conclusions. Experiments must have been conducted rigorously, with appropriate controls, replication, and sample sizes. The conclusions must be drawn appropriately based on the data presented.  **Reviewer #1: Yes**  **Reviewer #2: Partly** | Well noted. Thank you for very much for your kind feedback. |
| 2. Has the statistical analysis been performed appropriately and rigorously?  **Reviewer #1: Yes**  **Reviewer #2: Yes** | Thank you very much and much appreciated. |
| 3. Have the authors made all data underlying the findings in their manuscript fully available?  The [PLOS Data policy](http://www.plosone.org/static/policies.action#sharing) requires authors to make all data underlying the findings described in their manuscript fully available without restriction, with rare exception (please refer to the Data Availability Statement in the manuscript PDF file). The data should be provided as part of the manuscript or its supporting information or deposited to a public repository. For example, in addition to summary statistics, the data points behind means, medians and variance measures should be available. If there are restrictions on publicly sharing data—e.g. participant privacy or use of data from a third party—those must be specified.  **Reviewer #1: Yes**  **Reviewer #2: Yes** | Thank you and greatly appreciated |
| 4. Is the manuscript presented in an intelligible fashion and written in standard English?  PLOS ONE does not copyedit accepted manuscripts, so the language in submitted articles must be clear, correct, and unambiguous. Any typographical or grammatical errors should be corrected at revision, so please note any specific errors here.  **Reviewer #1: Yes**  **Reviewer #2: Yes** | Thank you very much for the kind feedback |
| **REVIEWER #1** | |
| **Comment 1** |  |
| I am not sure if your references and their citation in the manuscript follow the journal template or expectations. Your work needs minor modifications. Please consider citation of the following works: https://link.springer.com/chapter/10.1007/978-3-030-40417-8_9 and https://doi.org/10.1016/j.procs.2022.09.354  The factual figures and correctly performed inference and calculations are to be commended. | Thank you very much for the comment. PLOS ONE uses “Vancouver” style” for the references. Authors have revised the references to match the PLOS ONE format as below link.  <https://journals.plos.org/plosone/s/submission-guidelines#loc-reference-style> |
| **REVIEWER #2** | |
| **Comment 1** |  |
| The title needs rewording. The first part of the title suggests that the research is about how WFH changes work (that the research will have two states before WFH and after its introduction). What is not true. | Thank you very much. Well noted and revised the title to “Factors Affecting Job Performance of Sri Lankan IT Professionals Working from Home” – Line 25 to 27. |
| **Comment 2** |  |
| The abstract also needs rewriting. The beginning of the abstract is misleading as it suggests that the authors are addressing the issue of how WFH affects job performance. From the remainder of the abstract, it appears that selected factors affecting work-from-home performance are being investigated | Thank you very much for your feedback. Authors have rewritten the abstract to better describe the summary of the paper. This change is available in line 29 to 42.  “This study investigated the influence of physical work environment, work-life balance, work flexibility, and effective communication on the job performance of IT professionals in Sri Lanka's IT industry who work from home (WFH). A standard questionnaire was used to collect data from 293 IT specialists in 50 different IT organizations in Sri Lanka, and a stepwise probit model was used for data analysis. According to the findings, the physical work environment and work-life balance had a significant positive effect on job performance. A one-unit increase in physical work environment and work-life balance increased the likelihood of high job performance by 0.21% and 0.19%, respectively. In contrast, work flexibility had a negative effect on job performance, with an increase of one unit resulting in a 0.18% decrease in the likelihood of high job performance. The positive impact of effective communication on job performance was less significant. The study emphasises the significance of providing a conducive work environment and promoting work-life balance to improve the job performance of IT professionals in Sri Lanka's IT industry who WFH” |
| **Comment 3** |  |
| In the abstract, please include also 2-3 special quantitative achievements from the findings of this study. | Thank you very much for your comment and authors have added quantitative achievements into the abstract to better summarize the paper. This change is available in line 35 to line 39.  “…A one-unit increase in physical work environment and work-life balance increased the likelihood of high job performance by 0.21% and 0.19%, respectively. In contrast, work flexibility had a negative effect on job performance, with an increase of one unit resulting in a 0.18% decrease in the likelihood of high job performance. The positive impact of effective communication on job performance was less significant...” |
| **Comment 4** |  |
| The “Introduction” section needs a few more sentences to strengthen the article. Please add theoretical framework - indication of the specific theory on which the article is based | Duly noted and thank you very much. Authors have added the following paragraph to describe the theoretical framework of the study in the introduction section. This change is available in line 150 to 162.  “The theoretical framework for the study is based on the job demands-resources (JD-R) model, which suggests that job demands can lead to job strain and negative outcomes, while job resources can lead to job engagement and positive outcomes [14]. In this study, WFH is a job demand, whereas effective communication, work flexibility, work-life balance, and physical work environment are job resources. The study hypothesises that these resources can mitigate the adverse effects of WFH and boost job performance. Specifically, effective communication can reduce isolation and improve collaboration, physical work environment can provide a comfortable and conducive workspace, work flexibility can provide autonomy and control, and work-life balance can decrease work-family conflict and improve overall well-being. The JD-R model provides a theoretical lens through which the complex interaction between job demands and job resources, as well as how these can impact job performance in the context of WFH for IT professionals can be comprehended in Sri Lanka's IT industry.” |
| **Comment 5** |  |
| Please include the research problem, objective and questions in the last paragraph of the “Introduction”. | Thank you very much for your input. Authors have added the following paragraph to address this comment. This change is available in line 110 to 131.  “The COVID-19 pandemic has significantly altered how organisations operate, with remote work gaining popularity. Further, WFH policies have been implemented in worldwide to ensure business continuity and employee safety. Southeast Asian nation like Sri Lanka is not an exception to this trend. The country's thriving information and communications technology (ICT) sector heavily depends on remote work practices. However, little attention has been paid to how WFH affects the performance of IT professionals in Sri Lanka. Despite the abundant research on remote work, a focus on the performance of IT professionals during WFH is necessary, particularly in Sri Lanka. Filling this void, the present study focuses on the effects of effective communication, flexible work schedules, work-life balance, and physical work environment on the performance of employees during WFH. This study will be guided by the following research questions: How does effective communication affect employee performance during WFH?; How does a flexible work schedule impact WFH employee performance?; How does work-life balance impact the performance of employees during WFH?; and how does the physical work environment influence WFH employee performance?. By addressing these research questions, this study will contribute to gain a better understanding of the factors influencing the performance of IT professionals during WFH. Thereby, the study assists organisations and policymakers in Sri Lanka in strengthening their policy management and practices regarding remote work. This study also refers research work by multiple scholars during a 22-year timespan in 1998-2020 [4, 9-13].” |
| **Comment 6** |  |
| The description of the systematic literature review lacks details, e.g. the timeframe in which the articles were published, the languages, whether only peer-reviewed articles were considered, or if there were any further narrowing down. | Thank you very much for your kind observation. The authors have updated Figure 1 (box two) with the following content.  " …limited to January 1985 December 2021 (inclusive) and Peer Reviewed and English Language...” |
| **Comment 7** |  |
| The results presented in Figure 1 are not described in the body of the article. Please describe them. | Duly Noted and Thank you very much. Authors have updated the literature review section taking your inputs into consideration. Following is the added paragraph. This change is available in line 182 to 191.  “A total of 126 online publications with full text written in English language and peer reviewed were identified through the period from January 1985 to December 2021. The abstracts were then reviewed and discarded if: (i) these were irrelevant to the topic (n=21), (ii) these lacked sufficient information (n=15), or (iii) these were duplicates of previously published articles (n=35). This procedure led to the exclusion of 35 publications. Data were extracted from the remaining 55 publications and categorised into five subject areas: work-life balance (n=10), work flexibility (n=7), effective communication (n=12), physical work environment (n=8), and performance (n=18). To conclude the methodology, 55 empirical case studies were selected under the aforementioned five areas, and their key characteristics are summarised below.” |
| **Comment 8** |  |
| Please revise the literature review section. It is advisable that the paragraph titles correspond to the variables in the model. Please complete the section on the relationship of WFH to the physical work environment | Thank you very much for your kind observation. We have reviewed and expanded literature review to strengthen the relationship between work from home and physical work environment. This change is available in line 382 to 449.  “Working from Home and Physical Work Environment  When looking at the many elements that influence WFH, it was discovered that decreased interpersonal interactions, decreased management support and trust, and the appropriateness of the work atmosphere at home-office were highly important [46]. Workers who WFH can personalise their environment to meet their own specifications, requirements and preferences, which can improve job performance [47]. A study investigating the effect of WFH on job performance during the COVID-19 crisis in Indonesia stated that WFH allows workers to create a customised work environment tailored to their preferences and way of life [48]. Employee performance in WFH scenarios is positively influenced by a physically acceptable work environment, which has a substantial and favourable effect on performance. Having a well-maintained working environment with appropriate space, a peaceful ambience, good lighting, and improved working equipment enable employees achieve even greater levels of productivity [14, 49-51]. Additional studies indicated that workplace factors and operational commitment had an impact on job performance, both directly and indirectly when WFH, which support earlier results [52-54].  The Journal of Occupational and Environmental Medicine published a cross-sectional study on the performance of home-based workers in relation to their actual work environment in 2021. The study was conducted in Japan on workers who spent at least one day per month working from home. When working with WFH, the physical work space was a source of exposure. The Work Functioning Impairment Scale was used to assess the existence of work-related impairment. A "No" answer to suggested surroundings was shown to be a significant predictor of work functional impairment. Answering "No" to the question and "Is there enough light to perform my job?" were related to the greatest odds ratio of work functional impairment [28].  A paper published in 2020 disclosed that teachers' self-motivation in a remote work environment is influenced by various factors. These include their perceptions of the setting's ability to meet their psychological requirements for autonomy, competence, and interpersonal connection. To assist team leaders in being more responsive to the needs of their subordinates, the study have provided a list of practical tips [55] . Working from home requires a sense of trust, open communication, and a willingness to adapt to the needs of the team members. Their goal is to foster educators' self-motivation, which is useful not only for themselves but also for their colleagues and pupils at the institution. However, fewer research studies and articles relating the physical working environment to WFH policies have been published so far.  In conclusion, whether working in a traditional office or from home, the quality of the working environment can have a significant impact on employee morale and output. People who WFH anticipate a high-quality working environment with amenities such as sufficient equipment, privacy, and adequate lighting. Research indicates that a physical work environment supports an individual's work style, which has a positive effect on their telework performance. In 2020, a study discovered that the physical working environment has a significant positive effect on the performance of remote workers [56]. Consequently, it is hypothesised that a positive physical work environment has a positive effect on employee performance in a home-based work environment. |
| **Comment 9** |  |
| Hypotheses should follow directly from the literature. Please move them directly under the relevant sections with a description of the results of the literature review. | Thank you for your kind comment. Authors have revised the literature review and have added following for each sub section of literature review. These changes are available in line 289 to 294, line 317 to 325, line 375 to 381 and line 439 to 449.  **Working from Home and Effective Communication**  “…In summary, effective communication is essential for home-based workers, as it helps combat feelings of social isolation and disengagement. Clear communication regarding work expectations, job responsibilities, goals, objectives, and deadlines increases job satisfaction, loyalty, and productivity. Virtual communication is rife with miscommunication, which can result in less productivity during WFH. Consequently, it is hypothesised that effective communication has a positive effect on employee performance in a home-based office..”  **Working from Home and Flexible Work**  “…In conclusion, flexible work arrangements, such as WFH or working outside of normal business hours, are associated with a positive work attitude and high job performance. These arrangements assist workers in maintaining a healthy work-life balance, thereby enhancing job satisfaction and productivity. There is a correlation between flexible work arrangements and job performance, according to previous studies. The purpose of these arrangements is to assist employees in striking a balance between their personal and professional lives (i.e. work-life balance), which can lead to increased productivity. Therefore, it is hypothesised that flexible work arrangements improve employee performance in a home-based office.”  **Working from Home and Work Life Balance**  “…In summary behind the idea of "work-life balance" is that an individual's personal and professional lives should complement one another. It has been discovered that remote work has both positive and negative effects on work-life balance, which can affect worker performance and productivity. According to prior research, a lack of assistance in balancing professional and personal responsibilities can increase stress levels and result in subpar performance. Therefore, it is hypothesised that a healthy work-life balance has a positive effect on the performance of employees who WFH..”  **Working from Home and Physical Work Environment**  “…In conclusion, whether working in a traditional office or from home, the quality of the working environment can have a significant impact on employee morale and output. People who WFH anticipate a high-quality working environment with amenities such as sufficient equipment, privacy, and adequate lighting. Research indicates that a physical work environment supports an individual's work style, which has a positive effect on their telework performance. In 2020, a study discovered that the physical working environment has a significant positive effect on the performance of remote workers [56]. Consequently, it is hypothesised that a positive physical work environment has a positive effect on employee performance in a home-based work environment.” |
| **Comment 10** |  |
| Please separate the section on the specificity of WFH in the IT sector. | Duly Noted! We have added a section in the literature review called “Working from Home and IT Sector in Sri Lanka” to address this issue. This change is available from line 450 onwards. |
| **Comment 11** |  |
| Please provide detailed stages of the research process. | Thank you for your feedback. Authors have added “Figure 3: Flowchart of the Research Process” to explain the research process. This change is available in line 514. |
| **Comment 12** |  |
| Please add a table with the structure of the research sample. | Thank you very much for your feedback. Authors have added Table 4: Descriptive Statistics of Demographic Variables to explain the structure of the research sample. Furthermore, the following paragraph was added to explain the table. This change is available in line 608 to 620.  “Most respondents were men, indicating that the results may be skewed toward the male experience of WFH and performance in the research. In addition, a substantial proportion of the participants were married, and the majority of their spouses held employment. The majority of participants were college graduates and held engineering positions, with a significant proportion holding leadership positions within their respective organisations. Most participants were from enterprise organisations, while a smaller proportion were from Small Medium Enterprises (SMEs) and startups. Observations indicated that some organisations continue to use a hybrid model that combines office and remote work, while the majority support flexible work. The dependent and independent variables were derived using dummy variables, with 1 representing positive responses and 0 representing negative ones. The results are summarised in Table 5.” |
| **Comment 13** |  |
| Please add information about the research tools used, in particular where the questions came from (who formulated them and by whom they were validated beforehand) and on which scales they were answered. | Thank you very much for the feedback. Authors have addressed this with following paragraph. This change is available in line 498 to 511.  “To identify the factors that affect the performance of WFH, this research consulted the HR personnel from several well-known IT organizations in Sri Lanka and sought their opinion. This consultation was further supported by a literature review that examined previous studies related to the topic. The questionnaire, which served as the primary source of data for eliciting the perspectives of participants, was categorised into two sections. Section A focused on capturing employee demographics, whereas Section B focused on capturing the data necessary for the study's primary variables using Likert scale questions. The questionnaire is included in Appendix S1 of the supplementary materials. The Likert scale-based questions designed based on the findings of the literature review were pilot-tested with a sample of 30 participants, thereby eliminating two questions and modifying others to have a positive output. The pilot test demonstrated adequate reliability with a Cronbach Alpha value of 0.721, resulting in the distribution of the modified questionnaire to peers and networks as depicted in Figure 3.” |
| **Comment 14** |  |
| The inclusion of the model equation is not necessary. I suggest deletion. | Thank you for the comments. Authors used equation, since it can convey complex ideas and help to make scientific writing more precise and rigorous. By using equations, authors can clearly define the variables, parameters, and relationships involved in their work, which can help to ensure that their results are accurate and reproducible. Thus, authors would like to keep the model equation in the revised manuscript. |
| **Comment 15** |  |
| Please complete the description of the variables, e.g. what questions were included. | Thank you for your feedback. Well noted and we have added a table under data section with the title “Table 1: Operationalization of the Variables”. This change is available in line 540. |
| **Comment 16** |  |
| Please separate the “Results” section and please explain research problems, solutions, and the theoretical contribution of your study in the section. | Duly noted and thank you very much for your comment. Authors have separated Results and Discussion section in the revised manuscript address this issue. Results section is now available from line 630 to 700. Discussion section is available from line 702 to 775. These are two separate sessions in the revised manuscript. |
| **Comment 17** |  |
| In the section with “Conclusions” add paragraphs mentioning the limitations of the study and remedies to limitations. | Thank you for your feedback and we have added a section to address limitations to the revised manuscript. This change is available in line 804 to 811.  “Limitations  The study was conducted in 2022, at a time when the COVID-19 outbreak was ongoing. The uncertainty brought on by the pandemic has seen people suffering ample problems, triggering anxiety on whether people can continue living and upkeep their health. As a direct consequence of this, individuals did not answer to the surveys with much enthusiasm. The lockdown being in place at the time of the study has also proven to be a barrier in interacting with a greater percentage of the populace.” |
| **Comment 18** |  |
| Please add to the “Conclusions” section the future scope of your research. | Duly noted and we have added a paragraph to conclusion adding the future scope of the research. This change is available in line 791-803.  “This study prioritised increasing the sample size and heavily relied on quantitative data to meet the research objectives within the allotted timeframe. Nonetheless, this approach may have impeded in identifying a more nuanced comprehension of the diverse outcomes associated with remote work performance. Therefore, future research should adopt a mixed-method and qualitative approaches to gain a more complete understanding of the variables influencing remote work performance. In addition, the analysis of this study was limited to a set of factors including effective communication, work-life balance, a comfortable workplace, and flexible work assistance. However, to improve the validity of remote work policies, future research should investigate a broad range of variables that influence the efficiency of remote IT personnel. A more comprehensive approach that enables organisations to optimise their remote work policies and foster better work experiences for their employees, would be advantageous for both the employees and the organisation as a whole.” |
| **Comment 19** |  |
| Please correct typos in the text. | Thank you very much for your kind remarks and authors have rechecked and done a proofread on the revised manuscript. |
