# Supplementary material for: Factors affecting job performance of Sri Lankan IT professionals working from home
Source: PLoS One. 2023 Dec 20;18(12):e0295305. doi: 10.1371/journal.pone.0295305 (PMC10732380; doi:10.1371/journal.pone.0295305)
Supplement: Attachment — Submitted filename: Response to Reviewers.docx [file pone.0295305.s004.docx]

**Point by point response to editor and reviewers**

Dear editor and the reviewers,

We would like to express our profound appreciation to the editor and the reviewers for the valuable comments and suggestions made on our manuscript which were very helpful in revising and improving it.

Please note that the line numbers referred in this document is aligned with the revised manuscript which has track changes.

| **Comments of the Reviewer** | **Comments of the Authors** |
| --- | --- |
| **EDITOR** | |
| This paper lacks strong theoretical argument. When searching in databases, the authors missed many relevant papers (related, e.g., to telecommuting, virtual work, work from home, lockdown, Covid-19). Many articles have been published on the topic (job performance, telework) which referred to the Covid-19 pandemic. So, the research gap is not enough justified. You neither present the papers that undertook similar problems in your literature review nor compare their findings with your findings. Therefore, in my opinion, your contribution to science is not significant. Furthermore, the article which you used are not up-do-date. Please, enrich your paper by using relevant, high quality literature sources. | Thank you very much for your comment. This research was conducted in 2021/2022. We have added new references to the latest papers, including those from 2022 and 2023, in the most recent submission. We have also reviewed papers that reference the following query  Search Query: ("work life balance” or “virtual office” or “virtual platform” or “work from home” or “WFH” or “work life” or “telework’’) and ("communication” or “performance” or “efficiency” or “flexibility” or “balance” or “IT” or “Information Technology”)) limited to January 1985 March 2023 (inclusive) and Peer Reviewed and English Language.  As a research gap, we have identified the insufficient amount of research conducted on IT professionals in Sri Lanka regarding the effects of working from home on performance. We believe that the culture and mindset of people vary from country to country, emphasizing the need for conducting this research within the specific context of Sri Lanka.  In the original submission, we had 55 references, which we subsequently increased to 72 in the first revision. In the second revision, we further incorporated additional studies and increased the reference count to 87. |
| **REVIEWER #1** | |
| **Comment 1** |  |
| The study under review presents original research, but its rationale for conducting such research is not clear. The paper fails to provide convincing reasons to undertake this study, leaving the reader wondering about its significance. Additionally, it appears that some parts of the research have already been published in a previous study by the same authors, and the similarity between the two papers is too high. The reported results have already been made public, which raises questions about the originality and novelty of the study under review.  Despite these shortcomings, the study's experiments, statistics, and other analyses are of high technical quality. However, the paper's technical descriptions are inadequate and do not provide sufficient detail to replicate the experiments or verify the results. The references list is also a cause for concern as it has not been revised or extended. The absence of an updated references list casts doubts on the paper's scholarly rigor, and it may indicate that the authors did not conduct a thorough literature review. | Thank you for bringing to our attention the issue with the reference count not being updated in the submitted paper. We sincerely apologize for the oversight in not updating the reference number in Figure 1 during the previous iteration. The original submission indeed had 55 references, and the first revision included 72 references (17 new references). However, regrettably, the authors failed to update the reference count in Figure 1 for the first revision. In the second revision, we have included a total of 87 references (15 new references) and have rectified the reference count in Figure 1 accordingly.  The following references were added in the second revision.  29. Tooranloo, Azadi, & Sayyahpoor, 2017  30. Anakpo, Nqwayibana, & Mishi, 2023  31. Ahmad, Asmawi, & Samsi, 2022  40. Abdullah, Rahmat, Zawawi, Khamsah, & Anuarsham, 2020  41. Hamad et al. 2021  42. Kleine, Rudolph, & Zacher, 2019  47. Sousa-Uva et al., 2021  48. Grincevičienė, 2020  54. Pollitt, 2008  55. Raišiene, Rapuano, Varkulevičiute, & Stachová, 2020  57. Abioro, Oladejo, & Ashogbon, 2018  56 Golden, TDVeiga, & Dino, 2008  50. Ammons & Markham, 2004  1. Baker et al., 2006  59. Holtz & Harold, 2008  After conducting an extensive literature review, we have identified a research gap in the existing literature regarding the effects of working from home (WFH) on the job performance of IT professionals in Sri Lanka. In light of this gap, our research focuses exclusively on this particular group of professionals to investigate the impact of WFH on their performance. To the best of our knowledge, no previous studies have specifically addressed this research question within the context of Sri Lanka.  We would like to emphasize that our research differs from the study you mentioned in several key aspects. These distinctions encompass the sample coverage and data collection period, as well as the respondents and data analysis techniques employed. As a result, we believe our study makes a unique contribution to the field by addressing this specific research gap and providing fresh insights into the performance of IT professionals in Sri Lanka within the WFH context.  We hope that this clarification adequately addresses your question. If you have any further concerns or require additional information, please feel free to ask. We appreciate your positive comments on the analysis. Thank you. |
| **REVIEWER #2** | |
| **Comment 1** |  |
| Please correct some typos, e.g. in the keywords and section title there is "work life balance" (without hyphen) in the text "work-life balance" (with hyphen). I suggest alignment | Thank you very much. Well noted and revised the entire article to have “work life balance” |
| **Comment 2** |  |
| I did not notice in the body of the article when exactly (month and year) the questionnaire survey was conducted. Please add the information. | Thank you for your feedback. The authors have taken your suggestion into account and have now included the time period during which the survey was conducted. This change can be found in lines 395 to 398 of the revised paper with track changes.  “The questionnaire was conducted between April 2022 to October 2022. It was distributed through the company's social media accounts and personal contacts, utilising the internet as a means to reach IT professionals from the selected organisations. This distribution method allowed for the collection of primary data.” |
| **Comment 3** |  |
| Using the term 'impact' in hypotheses is usually risky. I would suggest replacing it with 'linked by a positive relationship'. | Thank you for your comment. The authors have made changes to the wording of the hypothesis based on your suggestions. This modification can be found in lines 478 to 485 of the revised paper with track changes.  “Hypothesis 1: Effective communication is linked with a positive relationship on employee performance in a home working environment.  Hypothesis 2: Flexible work arrangement is linked with a positive relationship on employee performance in a home working environment.  Hypothesis 3: Physical work environment is linked with a positive relationship on employee performance in a home working environment.  Hypothesis 4: Work life balance is linked with a positive relationship employee performance in a home working environment.” |
| **Comment 4** |  |
| I would suggest expanding the limitations section, e.g. in terms of the limitations of research methodology used | Thank you for your comment. The authors have included the following information in the limitation section, specifically in lines 778 to 785 of the revised paper with track changes.  “The study was conducted in 2022, at a time when the COVID-19 outbreak was ongoing. The uncertainty brought on by the pandemic has seen people suffering ample problems, triggering anxiety on whether people can continue living and upkeep their health. As a direct consequence of this, individuals did not answer to the surveys with much enthusiasm. The lockdown being in place at the time of the study has also proven to be a barrier in interacting with a greater percentage of the populace. The sample size of the study confined to Sri Lanka. Despite this limitation, the sample size was considered sufficient as it represented the population of IT professionals in Sri Lanka. However, for a more comprehensive understanding of the impact of working from home policies in the current era, future research should encompass other countries. Nonetheless, the findings of this study offer valuable insights into the experiences individuals in Sri Lanka during the pandemic. |
