# Supplementary material for: Factors affecting job performance of Sri Lankan IT professionals working from home
Source: PLoS One. 2023 Dec 20;18(12):e0295305. doi: 10.1371/journal.pone.0295305 (PMC10732380; doi:10.1371/journal.pone.0295305)
Supplement: Attachment — Submitted filename: Response to Reviewers.docx [file pone.0295305.s005.docx]

**Point by point response to editor and reviewers**

Dear editor and the reviewers,

We would like to express our profound appreciation to the editor and the reviewers for the valuable comments and suggestions made on our manuscript which were very helpful in revising and improving it.

Please note that the line numbers referred in this document is aligned with the revised manuscript which has track changes.

| **Comments of the Reviewer** | **Comments of the Authors** |
| --- | --- |
| **REVIEWER #2** | |
| **Comment 1** |  |
| In response to the reviews, the authors have emphasized the contextual significance focusing on the Sri Lankan workforce. Therefore, it is advisable to incorporate "Sri Lanka" as a keyword while eliminating the redundant terms "performance" and "employee performance." | Thank you for your valuable feedback. We have removed the keyword 'employee performance' and have added the keywords 'Sri Lanka' and 'Information Technology' to line 42. |
| **Comment 2** |  |
| The dynamics surrounding remote work are subject to rapid changes, with e.g. several global corporations presently advocating for a return to office-based work. Consequently, the introductory section may require an update or clarification to reflect the ongoing trend of expanding remote work in Sri Lanka. | Thank you for your thoughtful observation. This study was conducted in 2022 when the global trend favored remote work. However, as of 2023, there has been a shift towards office-based work. We have updated the introduction section in lines 100-103 to reflect this change:  “…It is worth noting that in 2023, organizations have started to shift back to office work policies, and WFH is not encouraged as much as it used to be. This study aims to provide better insight into that decision and explore what factors have affected work-from-home for IT professionals in Sri Lanka..…” |
| **Comment 3** |  |
| In the Introduction, it would be valuable to elucidate the necessity of conducting a distinct study on a Sri Lankan sample. Specifically, articulating the distinctive features of the Sri Lankan workforce that remain unexplored in previous studies conducted elsewhere would enhance the article's context. | Thank you for your valuable suggestion. We have incorporated a new paragraph in the introduction section (lines 106-118) to provide context for the study:  “In focusing on the Sri Lankan context, it is essential to highlight the unique circumstances and emerging status of the country in the realm of information technology. Sri Lanka, as a developing nation, presents a dynamic landscape where the IT sector is swiftly evolving. It is pertinent to recognize that Sri Lanka's IT literacy and workforce, in comparison to other developing countries, are factors that have remained unexplored in previous studies conducted elsewhere. For instance, exploring how Sri Lanka ranks in terms of IT literacy and its burgeoning IT sector compared to other developing countries can shed light on the distinctive features of the Sri Lankan workforce. According to the World Bank, Sri Lanka's IT sector has grown by 20% annually in recent years, outpacing the global average of 10%. The country's IT literacy rate is 60%, which is higher than other developing countries in the region, such as India (40%) and Bangladesh (25%) [14]. These factors, in conjunction with the global shift towards WFH, underscore the need for a focused examination of the performance of IT professionals in Sri Lanka during remote work scenarios.” |
| **Comment 4** |  |
| Recent investigations on productivity in remote work environments have revealed a decline in performance. For instance, studies indicate that employees assigned to full-time remote work exhibit an 18% decrease in productivity compared to their office counterparts. It is advisable to enrich the article with these contemporary findings. For additional information, refer to the study available at <https://www.nber.org/system/files/working_papers/w31515/w31515.pdf>. | Thank you for this valuable recommendation. We have enriched the literature review in lines 250-267 with the following additions:  “In a study done in 2017 with a sample size of 235 data entry workers in India, the authors explored how working from home affected worker productivity. They found that workers who were randomly assigned to work from home were 18% less productive than those who worked in the office. This difference was mostly due to the fact that workers who worked from home learned more slowly. The authors also found that workers who preferred to work from home were more productive than those who preferred to work in the office. However, this difference was not enough to offset the negative effects of working from home. Overall, the paper finds that working from home can have a negative impact on worker productivity. However, the authors also noted that there are some benefits to working from home, such as improved work-life balance and reduced commuting time [35]. In contrast, A study by the Sri Lanka Association of Software and Services Companies (SLASSCOM) found that Sri Lankan IT professionals performed well during remote work scenarios. The study found that 90% of IT professionals were able to maintain or improve their productivity while working remotely. Additionally, 85% of IT professionals reported that they were satisfied with their work-life balance while working remotely. Overall, the Sri Lankan IT sector is a dynamic and growing sector with a young and adaptable workforce [36]. IT professionals in Sri Lanka have performed well during remote work scenarios, demonstrating the country's potential to become a major player in the global IT industry.” |
| **Comment 5** |  |
| The section dedicated to "Working from Home and Flexible Work" encompasses various subtopics, including work-life balance (which is the subject of the subsequent section). The central theme of work flexibility may not be immediately evident. It is recommended to rephrase this section to ensure that the core concept of work flexibility is prominently highlighted. | Thank you for your suggestion. We have revised the section on 'Working from Home and Flexible Work' to emphasise the core concept of work flexibility. The changes are made in lines 349-382.  “Recent research illuminates the diverse dimensions of work flexibility and its implications for employees. In a study conducted in 2021, secondary school teachers in Ekiti State displayed a modest level of work-life balance, work flexibility, and job efficiency, underscoring the interconnectedness of work-related stress, workload, and job performance. Work-flexibility, in this context, significantly influenced work life balance, which in turn, influenced the teachers' job performance. Furthermore, insights gained from investigations into remote work experiences during the initial wave of the COVID-19 pandemic in Portugal underscored the impact of the work environment and flexible work culture on telework happiness [50]. Comprehensive research is required to further understand telework satisfaction and its effects on physical and mental health, facilitating the development of effective strategies for enhancing employee well-being and cultivating a conducive teleworking environment. As the scope of telework and mobile work arrangements continues to expand due to advances in digitalization, it reshapes working conditions and job quality. A study published in the International Journal of Environmental Research and Public Health delved into the impact of various telework forms on job quality. The research revealed that gender, the nature of telework, flexible hours in telework and the intensity of information and communication technology (ICT) usage significantly shape working conditions and job quality [51]. Notably, home-based teleworkers who regularly work from home reported the highest job quality, underscoring the benefits of work flexibility. However, this achievement sometimes comes at the cost of lower skill development, decision-making autonomy, pay, and career opportunities. This research aims to investigate current data on job quality and work flexibility, with a particular focus on gender as a critical aspect of analysis. Regarding employee performance, a flexible work arrangement was found to have no direct impact.  As the demands of both professional and personal life grow, so does the need for effective time management and work overload. An individual's ability to function well at work is adversely affected by factors such as job overload and poor time management. In a study published in the Dynamic Relationships Management Journal [52], researchers looked at how time management affects job performance and the link between work overload and flexible working hours which lead to work life balance. The findings revealed that effective time management reduces the negative effects of work overload and poor job performance when combined. It means workplace productivity and work–life balance suffers because of overwork. Considering these results, it is essential for both people (employees) and companies (employers) to pay greater attention to time management to promote work–life balance and productivity.” |
| **REVIEWER #3** | |
| **Comment 1** |  |
| In the introduction section it is written "The theoretical framework for the study is based on the job demands-resources (JD-R)". Can this be clarified in more depth so that it does not become ambiguous, or can it also be expanded in the literature section. | Thank you for your valuable feedback. To provide a clearer understanding of the theoretical framework, we have included a paragraph in the literature review (lines 160-184) that elaborates on the Job Demands-Resources (JD-R) model.  “The theoretical underpinning of this study is grounded in the Job Demands-Resources (JD-R) model, an influential framework in the field of organizational psychology and work-related research. Developed to examine the interplay between workplace elements and employee well-being, the JD-R model posits a fundamental distinction between two categories of factors: job demands and job resources [15]. Job demands encompass the aspects of work that require sustained physical or psychological effort and are associated with the potential for strain and negative outcomes. In the context of this study, the act of WFH serves as a pertinent job demand, given its potential to introduce challenges and stressors into the professional lives of IT professionals in Sri Lanka. On the other side of this theoretical spectrum are job resources, which are the elements of work that facilitate the achievement of work goals, reduce job strain, and promote positive outcomes. In this study, we identify four critical job resources that play a pivotal role in enhancing the job performance of IT professionals during WFH: effective communication, work flexibility, work-life balance, and the physical work environment. Effective communication fosters collaboration and alleviates the isolation often associated with remote work. Work flexibility empowers employees with autonomy and control over their work schedules and conditions. Work-life balance addresses the delicate equilibrium between personal and professional life, a crucial determinant of overall well-being. The physical work environment, even in the context of remote work, provides a comfortable and conducive workspace for IT professionals. The JD-R model serves as an invaluable theoretical lens through which the complex interplay between job demands and job resources is examined. It offers a comprehensive perspective on how these factors can influence job performance in the unique setting of WFH for IT professionals within Sri Lanka's IT industry. Through this framework, this study seeks to unravel the intricate dynamics of remote work and offer insights that can guide organizations and policymakers in enhancing work conditions and performance for IT professionals in an evolving work landscape [15].” |
| **Comment 2** |  |
| Of all the existing findings, the discussion section does not clearly explain the novelty and value of the contribution (Need a special paragraph). | Thank you for your valuable feedback. In response to your suggestion, we have included the following paragraph in the discussion section (lines 751-774) to highlight the novelty and significance of this study.  “This study's significance extends beyond the boundaries of traditional research on remote work and job performance. While existing literature has primarily focused on developed economies and their well-established information technology sectors, this research probes into the intricacies of WFH within the context of a developing nation. Sri Lanka's burgeoning information and communications technology (ICT) sector, though promising, faces unique challenges and opportunities. As developing countries like Sri Lanka make strides in the IT industry, they encounter distinct scenarios that demand an in-depth investigation. The IT professionals operating in these regions often navigate a complex landscape, where innovation and resourcefulness are essential to address the diverse demands of clients from across the globe. The practicality and adaptability of remote work, as explored in this study, emerge as vital determinants of their effectiveness. Furthermore, the study underscores the specific nuances of IT work in a developing country, where adaptability, self-reliance, and creative problem-solving skills are paramount. Unlike standardized, repetitive tasks found in certain sectors, the IT domain demands adaptability and innovation to tackle multifaceted issues. The results of this research spotlight how these qualities, often intrinsic to IT professionals in such settings, interact with the components of remote work.  By illuminating these interactions and their impact on job performance, this study offers not only a deeper understanding of remote work but also practical insights for emerging economies. As the global workforce evolves, bridging the gap between established practices and novel challenges is critical. The significance of this research lies in its potential to inform strategies for managing remote work in developing countries, thereby contributing to the robustness and adaptability of their IT sectors. The findings provide a valuable resource for policymakers, organizations, and IT professionals alike as they navigate the ever-evolving landscape of remote work.” |
| **Comment 3** |  |
| For future research there are no suggestions regarding methodological aspects. | Thank you for your feedback. To provide more insight into future research, we have included Cross-Cultural Variations, Multinomial Analysis, Stressors and Mental Health, Digital Tool Adoption and Comparative Analysis. These research directions hold the potential to inform more adaptable and culturally sensitive remote work policies and practices for IT professionals and organizations in the future. This can be seen in the revised manuscript in lines 869-884:  “While this study provides valuable insights into the dynamics of WFH among IT professionals in Sri Lanka, there are several promising avenues for future research to further enrich our understanding of remote work in the ever-evolving landscape. Firstly, exploring cross-cultural variations in remote work experiences can unveil how cultural nuances shape the effectiveness of WFH policies, leading to more tailored approaches for different regions. Secondly, conducting multinomial analysis of employee performance, particularly by breaking down results based on distinct job designations, can provide a more nuanced understanding of how remote work impacts IT professionals at different career stages and roles. Thirdly, an examination of the specific stressors, coping mechanisms, and the impact on mental health among IT professionals working from home could offer critical insights for enhancing remote work policies. Additionally, studying the adoption of emerging digital tools and platforms in the remote work setting can provide insights into the changing technological landscape of the industry. Lastly, a comparative analysis between pandemic and post-pandemic remote work environments can reveal enduring adaptations and evolving trends in the realm of remote work. These research directions hold the potential to inform more adaptable and culturally sensitive remote work policies and practices for IT professionals and organizations in the future.” |
